# Absorption avoided resonance crossing of hybridization of silicon nanoparticles and gold nanoantennas

**DOI:** 10.1038/s41598-019-48135-y

**Published:** 2019-08-13

**Authors:** Jhen-Hong Yang, Min-Wen Yu, Kuo-Ping Chen

**Affiliations:** 1 Institute of Photonic System, College of Photonics, National Chiao Tung University, Tainan, Taiwan, ROC; 2 Institute of Lighting and Energy Photonics, College of Photonics, National Chiao Tung University, Tainan, Taiwan, ROC; 3 Institute of Imaging and Biomedical Photonics, College of Photonics, National Chiao Tung University, Tainan, Taiwan, ROC

**Keywords:** Nanoparticles, Polaritons

## Abstract

The near-field coupling between a high-refractive-index nanoparticle and gold nanoantennas is investigated theoretically. The absorption enhancement and also avoided resonance crossing in the absorption cross section spectra were observed with the hybridization system due to the coupling between the localized surface plasmon resonance of the gold nanoantennas and the magnetic dipole resonance of the silicon nanoparticle. By controlling the nanoparticle size or the separation distance, the near-field coupling can be tuned from the weak to the strong regime.

## Introduction

The hybridization of nanoantennas (NAs) has attracted much attention recently. There has been research involving not only metal NAs, but also the hybridization of heterostructures such as NAs with microcavities^1,2^, NAs with dielectric particles^3–5^, and Janus dimer antennas^6–9^. The hybridization of heterostructures are also applied in nonlinear optics^10,11^, and unidirectional scattering^12–14^. When localized resonance occurs in the spherical nanoparticles, the scattering and absorption cross-sections can be calculated by Mie theory^15^. Both the metal and high-refractive-index (HRI) nanospheres can be analyzed in detail by using the same mathematical framework. In metal nanoparticles (NPs), localized surface plasmon resonance (LSPR) as a collective oscillation of conduction band electrons could be excited by the incident light^16,17^. In HRI NPs, the fundamental electric/magnetic type resonance is called electric dipole (ED)/magnetic dipole (MD) resonance^9,13,18^. Recently, the combination of gold NAs with ED and MD of dielectric NPs has been applied to various applications such as biosensors^4,19,20^, water splitting catalysts^3^, and Purcell enhancements^2,21^. In general, the common structure design of a dielectric resonator is in the form of a disk^2,22,23^, cylinder^1^, or sphere^4,19,20,24,25^. Because of the progress in nanotechnology, the dimensions of disks and spheres can now be shrunk to nanometer sizes.

In applications of hybridization of gold NAs with ED and MD^2–4,26^, the electric field enhancement is the most important criterion, and the absorption efficiency is usually proportional to the strength of the localized electric fields^27–29^. However, there are still few studies discussing the interaction between gold NAs and ED/MD of HRI NP. In this work, the unique interaction between LSPRs and HRI-ED/MD resonances has been studied. A strong absorption enhancement, which is due to the coupling between HRI-ED/MD and LSPRs, has been demonstrated and the possible avoided resonance crossing^30,31^ is studied numerically in the hybridization system.

**Figure 1:**
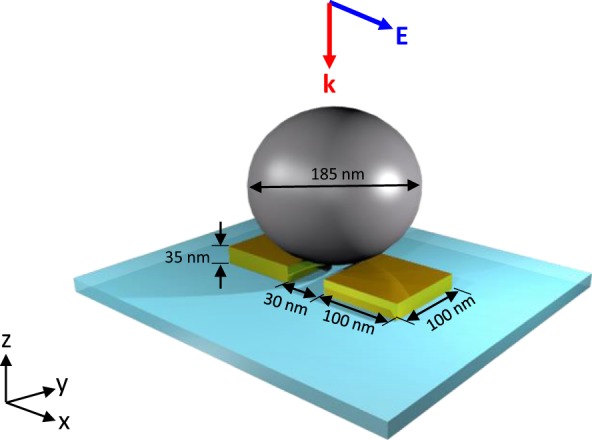
The schematic of hybridization system with a silicon NP and gold NA. The width, thickness and gap length of NA are 100 nm, 35 nm and 30 nm respectively. The diameter of silicon NP is 185 nm. The substrate is glass (n = 1.52). The plane wave is illuminated in the negative z-direction, and the electric field is along the x-axis.

**Figure 2:**
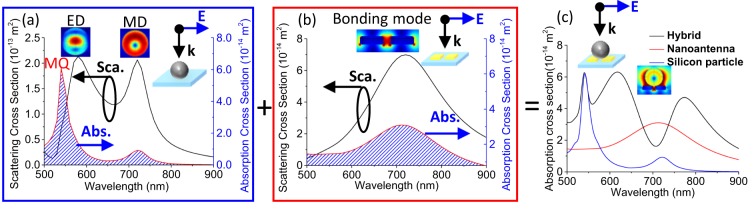
Scattering and absorption cross-sections of (**a**) silicon NP and (**b**) gold NAs. (**c**) Comparison of the silicon NP, gold NAs, and hybrid structure in terms of the absorption cross-section. The inset of each figure displays the near-field distribution of each resonance mode. The width, thickness and gap length of NAs are 100 nm, 35 nm and 30 nm respectively. The diameter of silicon NP is 185 nm. The substrate is glass (n = 1.52).

The schematic of the hybridization system has been shown in Fig. 1. The reason to choose paired gold NAs is due to the stronger localized electric fields and the broader resonance bandwidth comparing to a single gold NA^32^. To avoid the singular resonance of silicon particle, the rounded sphere was used in our study. In Fig. 2a,b, the scattering and absorption cross-sections of the silicon NP and gold NAs are shown, respectively. The two absorption resonance peaks in Fig. 2a are due to the MD and magnetic quadrupole (MQ) resonances and the intrinsic loss of the silicon particle. The magnetic-type resonance exists in the HRI NP because the resonance acts as a cavity mode, which implies that the energy is trapped in the particle; thus, the magnetic-type resonance is not sensitive to the variation in the medium where the particle is embedded. In contrast, the electric-type resonance does not exist any intrinsic absorption because the electric field are spreading out the particle; thus, the electric-type resonance is sensitive to detect the variation of surrounding medium. Once the electromagnetic waves have been trapped in the particle (cavity), the intrinsic absorption of the material is enhanced, as shown in Fig. 2a. In Fig. 2b, the resonance of the gold NAs would result in strong absorption and scattering at around 710 nm because of the LSPR. As shown in the inset of Fig. 2b, the electric field is enhanced in the gap of NA. The absorption cross-sections of the NAs, silicon NP, and hybrid structure are shown in Fig. 2c, and the near-field distribution at 710 nm is shown in the inset.

**Figure 3:**
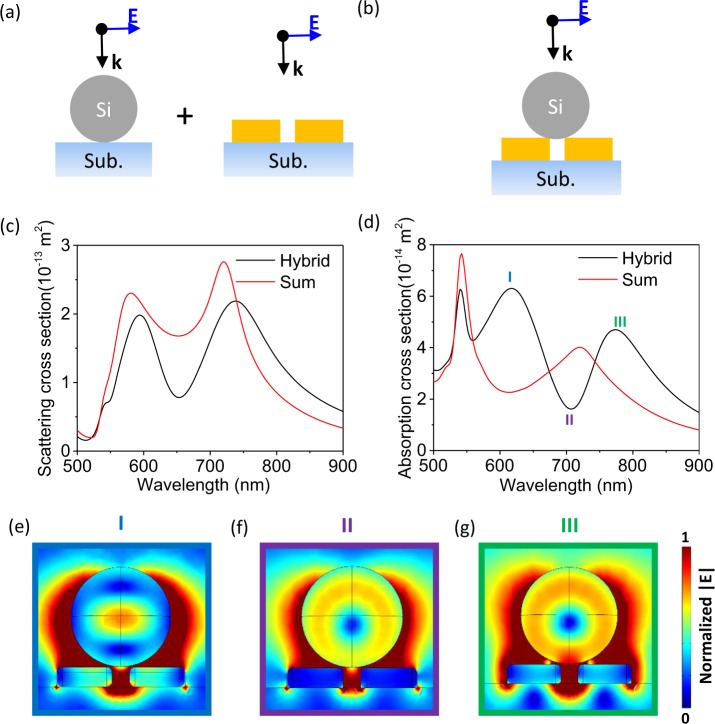
The schematic of (**a**) summation and (**b**) hybrid system. Comparison of (**c**) scattering cross-section and (**d**) absorption cross-section for the hybrid structure (black line) and summation of the silicon NP and gold NAs (red line). The Roman numbers, I, II, and III in (**d**) correspond to the near-field distributions of the hybrid structure at (**e**) 620 nm, (**f**) 710 nm, and (**g**) 760 nm. The scale of near-field distributions are normalized to the maximum field intensity.

In order to further study the hybridization effect numerically, the finite element method (FEM) was used in the simulation. The FEM model is set up by two electromagnetic wave modules. The first module is utilized to establish the background plane wave distribution. Then, in the second module, the scattering energy is integrated by removing the influence of background. In the simulation, the excitation source is a plane wave, which is incident in -z direction in Fig. 1. To simulate the extinction cross section of the single hybridization structure, all the boundaries in FEM are perfect matching layer (PML) for absorbing the reflected light. Figure 3a,b show the schematic of the summation and the hybrid system. In Fig. 3c,d, the scattering and absorption cross-sections are compared for the hybridization of the gold NAs and HRI NP against the summation of the gold NAs and HRI NP. In Fig. 3c, it is evident that the superposition characteristic is exhibited in the scattering cross-section. However, the absorption cross-section clearly indicates a different characteristic between the hybrid and summation results. In Fig. 3d, the absorption cross-section shows an avoided resonance crossing dip in the hybrid system instead of the summation result, which indicates that a strong interaction exists when the gold NAs and silicon NP are hybridized with each other. The reason of the avoided resonance crossing dip could not be zero is because of the coupling efficiency is not strong enough in this hybridization system^33^. The near-field distributions of the resonance peaks and dips in Fig. 3d are shown in Fig. 3e–g in different colored boxes, which correspond to the color of the Roman numbers in Fig. 3d. By comparing Fig. 3e–g, as well as the black line in Fig. 3d, it is observed that the electric fields inside the NAs are strong at 620 and 760 nm (peaks of black line in Fig. 3d), which implies that the resonance of the NAs is enhanced when the silicon NP is closer to the NAs. The distinct enhancement in Fig. 3d is due to the Mie resonance^3^. In our previous research, the absorption cross-section could be enhanced by evanescent waves^34^. In contrast, the electric fields inside the NAs are weak at 710 nm (dip in black line in Fig. 3d), which implies that the resonance of the NAs is suppressed at the resonance wavelength of the MD; the electric field is stored in the particle. In Fig. 3e–g, the electric fields outside the particle and NAs are always strong at the three resonance wavelengths, which implies that the dip at 710 nm is not due to the detuning effect, because the coupling is still strong at the wavelength of the dip. Comparing with absorption and scattering cross-section spectra from Fig. 2, the value of absorption cross section in MD and LSPR are close to each other, so the interference (avoided resonance crossing) would be apparent (Fig. 3(d)). In contrast, the scattering cross section of silicon NP and gold NAs are not in the same order, which means that the interference phenomenon would be not obvious. Because the scattering cross section of silicon nanosphere is much larger than that of gold NAs, the scattering signal would be dominated by the silicon nanosphere, and the spectra would not be affected much after hybridization (Fig. 3(c)).

**Figure 4:**
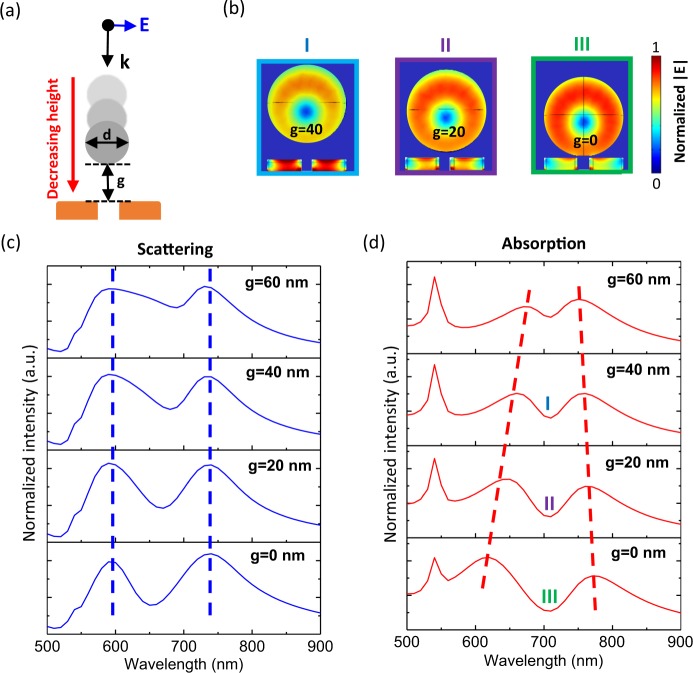
(**a**) Schematic of hybrid structure simulation and the corresponding (**c**) scattering cross-section and (**d**) absorption cross-section with different distances between the gold NAs and HRI NP. (**b**) Near-field distributions at 710 nm with different distances between the gold NAs and HRI NP. The numbers (I–III) in (**d**) correspond to the near-field distributions (**b**) of the hybrid structure of different distances. The scales of near-fields are normalized to the maximum field intensity.

By modifying the distance between the gold NAs and silicon NP, the hybridization of the LSPR and HRI-ED/MD can be analyzed. In Fig. 4a, different distances (g) between the NAs and silicon NP are shown. By comparing Fig. 4c,d, it is apparent that the resonance peak and magnitude in the scattering cross-section spectrum does not change with the different distance (g). The resonance dip in the absorption cross-section spectrum is fixed at about 710 nm, and the separations between the two peaks increase as the distance (g) decreases, which makes a description of the two peaks in the absorption are not related to the ED and MD resonance peaks in the scattering. The distance between the NAs and silicon NP was altered to verify the existence of avoided resonance crossing in the absorption. In Fig. 4d, it can be observed that the absorption cross-section represents the destructive interference when the absorption peak of the silicon NP is close to that of gold NAs. In Fig. 4b, the near-field distributions of the NAs at 710 nm (wavelength of dip in Fig. 4d) are shown with different distances (g). The electric fields inside the NAs are stronger when the silicon NP is away from the NAs, which denotes that the silicon NP seizes the energy if the silicon NP and NAs are sufficiently close to each other.

The mechanism behind the avoid resonance crossing is the interference between LSPR and MD. The interaction between NAs and NP should be near-field coupling because the distance (g) is much shorter than the resonance wavelengths. When the distance (g) is increasing, the mode coupling of silicon NP and gold NA become weak. Therefore, NP and NAs should be treated as two independent objects, which is the reason why the scattering and absorption spectra are the superposition of NAs and NP. For strong coupling with a short distance (g), the modes of NP and NA would interfere with each other. Due to the strong coupling, the NP and NA should be treated as one object, which also means that the resonance of these two objects are not independent. The split absorption peak (Fig. 4(d)) and continuity field distribution (Fig. 3(e–g)) between NP and NA are the evidence of strong interference (near-field coupling).

**Figure 5:**
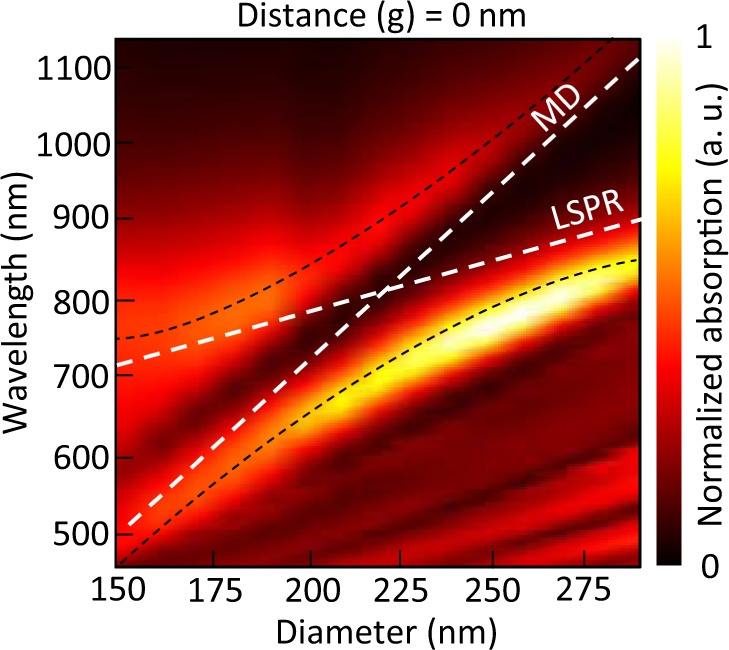
The color mapping of absorption spectra with varying diameters of silicon nanoparticles. The distance (g) = 0 nm. The dispersions of MD and LSPR are shown in white dash lines, and the black dash lines show trends of the split absorption peaks.

Figure 5 displays the absorption cross-section spectra of different silicon NP sizes. By changing the diameter of the silicon NP, the resonance wavelengths of MD and LSPR would shift. The apparent mode splitting could be observed where the MD and LSPR are crossing. When the avoided resonance crossing happens, the absorption peak of MD would transform into dip, and the absorption enhancement peak would be split, which is also shown in Fig. 4d.

In summary, the hybridization of gold NAs with HRI NPs has been analyzed in this work. The intrinsic absorption of HRI NP and the absorption enhancement of hybrid systems have been demonstrated by simulations at visible wavelengths. The numerically characterization of avoided resonance crossing have been verified for different silicon particle sizes. Interestingly, avoided resonance crossing did not occur in the scattering but in the absorption when the LSPR overlapped with the MD resonance. Similar effects have been demonstrated in the bimetallic heterodimers^35,36^, but less discussed in HRI NPs and gold NAs hybridization systems. The absorption in the metallic NAs and dielectric NP hybrid structure was enhanced compared to that of the gold NAs only, and surprisingly, most of the absorption was contributed by the NAs. We believed that our findings can be applied to the design of plasmonic photocatalysts with the metal-dielectric hybrid structure.
